# UAV-Based Intelligent Transportation System for Emergency Reporting in Coverage Holes of Wireless Networks

**DOI:** 10.3390/s21196371

**Published:** 2021-09-24

**Authors:** Abdullah M. Almasoud

**Affiliations:** Department of Electrical Engineering, Prince Sattam Bin Abdulaziz University, Al-Kharj 11942, Saudi Arabia; am.almasoud@psau.edu.sa

**Keywords:** unmanned aerial vehicle (UAV), emergency, emergency dispatcher, coverage holes, path loss map

## Abstract

During critical moments, disaster and accident victims may not be able to request help from the emergency response center. This may happen when the victim’s vehicle is located within a coverage hole in a wireless network. In this paper, we adopt an unmanned aerial vehicle (UAV) to work as an automatic emergency dispatcher for a user in a vehicle facing a critical condition. Given that the UAV is located within a coverage hole and a predetermined critical condition is detected, the UAV becomes airborne and dispatches distress messages to a communication network. We propose to use a path loss map for UAV trajectory design, and we formulate our problem mathematically as an Integer Linear Program (ILP). Our goals are to minimize the distress messages delivery time and the UAV’s mission completion time. Due to the difficulty of obtaining the optimal solution when the scale of the problem is large, we proposed an efficient algorithm that reduces the computational time significantly. We simulate our problem under different scenarios and settings, and study the performance of our proposed algorithm.

## 1. Introduction

With the rapid advancement in communications and electronics, unmanned aerial vehicles (UAVs) have emerged as promising enablers for the next generations of wireless networks [1,2]. UAV communications open the door for the innovator and the entrepreneurs to come up with disruptive solutions and a massive number of beneficial applications [3]. The applications of the UAV communications include (1) coverage extension for communication networks after disasters, (2) message relying between Internet of Things (IoT) devices, and (3) dispatching distress messages from a device located within a coverage hole to the emergency center.

In [4], the authors considered a joint optimization for resource allocation and UAV deployment in emergency scenarios. They proposed a scheme for recovering and maintaining the network connectivity in disasters. The work in [5] presents a UAV serving a group of users with different quality of service requirements in an emergency. The UAV supports the downlink for the users while optimizing its 3D location and power and bandwidth allocations.

In [6], a framework for UAV-assisted emergency network is proposed based on collaborated multi-UAVs. To achieve the goal, the trajectory and scheduling of the UAV are optimized. Moreover, a multi-hop device-to-device communication is established to extend the coverage of the network. The UAVs relay the information from the disaster area to an emergency communication vehicle. A UAV-assisted Wi-Fi network is proposed in [7] to help the personnel in the relief centers to gather surveillance information. The proposed network aims to expedite the rescue operations and provide an updated information about the disaster. UAV-assisted emergency communication is employed in a heterogenous IoT in [8]. The authors proposed a multi-objective resource allocation scheme to accommodate the surviving users and IoT devices during disasters to quickly recover the connectivity to them.

In [9], the authors considered an energy-efficient scheme used by cooperative UAVs that collect and disseminate data. Given that a set of fields are located within coverage holes, a cooperative set of UAVs collect data from these fields and cooperate in delivering the collected data to prolong the lifetimes of the UAVs. A group of UAVs can meet at a certain way point and send their messages to only one UAV. Then, the UAV that received the collected data from that group of UAV proceeds its flight to a communication network to deliver the messages or meets another cooperative UAV at a way point. After the UAV delivers the message to another UAV at a way point or delivers it directly to a communication network, the UAV returns to its field. In [10], a data dissemination scheme is proposed to disseminate data to a set of IoT devices using a UAV. The UAV is equipped with a cognitive radio, and it accesses a licensed band opportunistically when it is idle.

It is shown in [11] that a group of UAVs can be used to detect incidents on the roads of the urban areas. The UAVs provide the rescue teams with helpful information to expedite the rescue process. A robust routing scheme is introduced to guarantee communication stability during the process of delivering the distress information. A novel UAV path planning frame work is introduced in [12] for emergency messages transmission and collection. The motion and transmission power of the UAV is optimized while visiting access points for sending and collecting the emergency messages.

UAVs are considered essential enablers for intelligent transportation systems in the smart city [13,14]. The next generation of intelligent transportation systems requires integration of connected and autonomous vehicles. UAVs support the other vehicles on the ground by providing wireless network connectivity in an efficient way. It is shown in [13] that UAVs can work as flying accident report agents, flying roadside units or flying police eyes. In [14], a generic management framework for the UAVS in intelligent transportation systems is proposed. The authors investigated the UAV communication coverage in intelligent transportation systems. Moreover, they studied the problem of charging and docking the UAVs in the intelligent transportation systems.

UAVs can be used as aerial base stations for public safety applications. In [15], the authors studied the deployment of the UAVs as a part of the heterogeneous network when the network infrastructure is damaged. Then, the UAVs are utilized in public safety communication during disasters such as earthquakes. The authors analyzed the throughput achieved when utilizing the UAVs, and they proposed a genetic algorithm that optimizes the locations of the UAVs while supporting the public safety communication. The work in [16] presented the importance of incorporating the UAVs in heterogenous networks for supporting the public safety communication. It is shown that the UAVs can improve public safety communication by extending the network coverage and capacity.

In [17,18], UAV-based disaster-resilient architectures are presented. The proposed architecture in [17] consists of three layers: (1) a Software-Defined Networking (SDN) layer for centralized control, (2) UAV cloudlet that enables emergency communication, and (3) A radio access layer. This architecture supports delay sensitive applications such as the applications of emergency response systems. In [18], a distributed and expansible architecture for emergency communications is proposed. The area affected by disaster is divided into numerous sub-areas. Then, a UAV is assigned to each sub-area to help relay the emergency message to a communication network.

In [19], a scheme is proposed which utilizes sensors to detect a predetermined condition and a UAV to convey an emergency message to the closest communication network. The UAV is attached to an object, and it becomes airborne when a predetermined condition is detected. When the UAV is located within a coverage hole, it flies to the closest network access and sends the distress messages. In this paper, we investigate the proposed scheme by modeling the system, formulate the problem mathematical and optimize the delivery time for the distress message and the mission completion time for the UAV.

**Figure 1:**
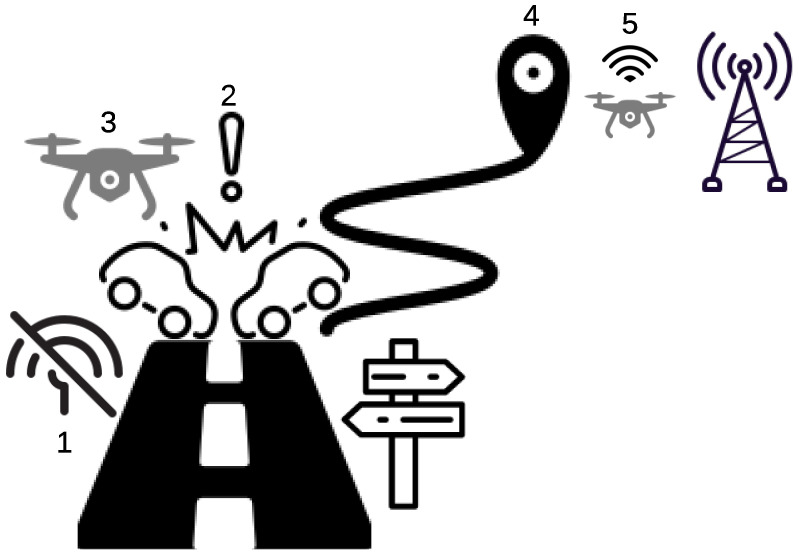
The UAV conveys emergency messages and sends them to the closest available network access point.

We assume in our work that the UAV is attached to a vehicle, and it detaches from it when a triggering event happens, such as a rapid and sudden change in the acceleration of the vehicle. Once an accident is confirmed, the UAV checks to see whether it is located within a coverage hole or not. If it is located within a communication network, it sends a distress message directly to the emergency response center. Otherwise, it flies to a location where a communication network is accessible and sends the distress message accordingly. Figure 1 shows an application of our proposed solution. First, two vehicles enter a coverage hole area where the communication service is not available. Second, an excessive change in the vehicle acceleration is detected by the UAV. Third, the UAV detaches itself from the vehicle right after the prediction of the accident. Forth, the UAV searches for the closest location with an access to a communication service once the collision is confirmed. Finally, the UAV sends the distress messages to the emergency center.

### Motivations and Contributions

It is not unusual nowadays that some people die because they face a problem with their vehicles outside the urban area. They may not be able to ask for help due to unavailability of the communication services. The problems that the traveler’s vehicle may face include accidents, battery discharging, running out of gas, being stuck in sand dunes, failure of the Global Positioning System (GPS) and many other problems.

It is reported in [20] that a couple died of thirst in a Saudi southern desert, where something went wrong with their vehicle. They could not ask for help due to unavailability of the cellular communication in the vast empty desert. Moreover, it is reported in [21] that three persons died near an outback community in central Australia. Their car broke down and they could not call for emergency due to unavailability of the cellular coverage. Even when the communication services are available in general, small coverage holes are still an issue in some areas such as valleys. When an accident happens in a coverage hole, an automatic emergency message dispatcher is needed to save the lives of the people. Accordingly, we proposed a UAV-based system to detect the accident of the vehicle and dispatches emergency messages automatically (or manually if needed) to the emergency center.

The contributions of our paper can be summarized as follows.

- We propose the path loss map to model the uplink transmission of the UAV and the trajectory design.
- We model the problem of sending the distress messages from the location of the accident to the communication network when the accident happens within a coverage hole.
- We propose an algorithm that solves our formulated problem more efficiently and provides a solution while reducing the computational time significantly.
- We simulate our problem and study the performance of our proposed algorithm under different scenarios and setting.

The rest of the paper is organized as follows. We describe the system model in Section 2, then we formulate our optimization problem in Section 3. Section 4 presents the proposed algorithm to solve our problem more efficiently. Finally, we shows the simulation results in Section 5, and conclude our paper in Section 6.

## 2. System Model

We consider a vehicle located outside the coverage of the communication services. This vehicle faces an accident, and it requires sending distress messages to the emergency center. Inspired by the work in [19], the UAV is attached to the vehicle and equipped with acceleration sensors. The UAV keeps measuring the acceleration while it is attached to the vehicle. Once the acceleration of the vehicle reaches a threshold π, the UAV detaches itself from the vehicle. Then, the UAV goes to the closest location with communication services and sends the distress messages. These messages include information such as the location of the accident, video, images, sounds and sensor data related to the accident. Once the UAV sent the distress messages, it will go to its predefined final location.

**Figure 2:**
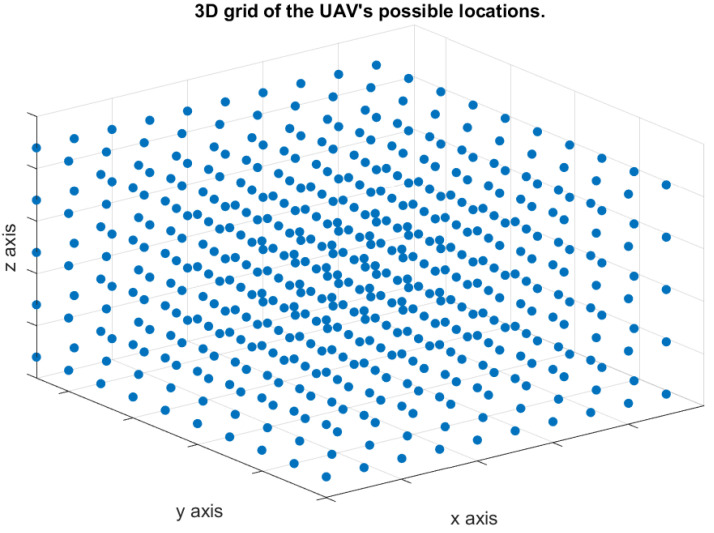
Three-dimensional path loss map.

**Figure 3:**
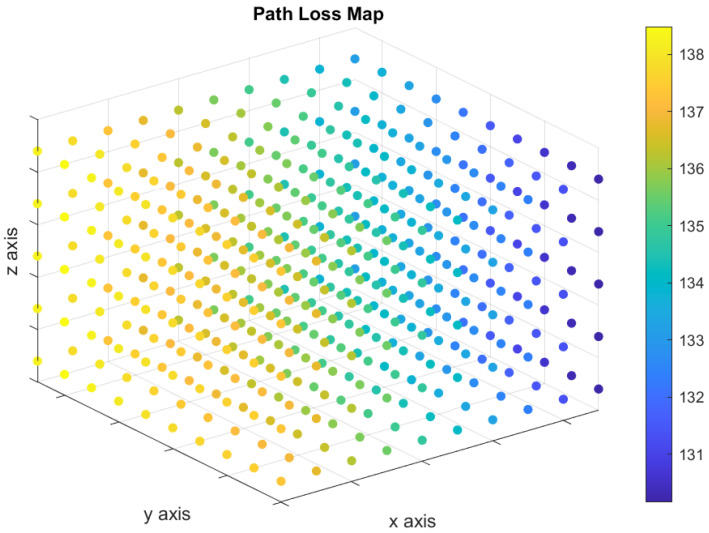
Colored path loss map indicating the path loss values (in dB) associated with each point.

The UAV moves in a three-dimensional space to convey the distress messages. Therefore, there are infinity points in the three-dimensional space can be used to represent all possible locations of the UAV during each time slot. Consequently, we discretize the three-dimensional space, as shown in Figure 2, to make our model practical. We define the “Path Loss Map” to be a three-dimensional map showing a set of points in a three-dimensional space, and each point is associated with a path loss value as shown in Figure 3. The path loss map consists of *N* points, and each point *i* is associated with a location on the three-dimensional space, ($x_{i},y_{i},z_{i}$), in addition to the path loss value. The number of access points is *B*, and the path loss value at any location is associated with one access point that results in the minimum path loss value. The color of each point in the path loss map indicates the path loss value in dB as shown in the colored scale.

The UAV uses the path loss map to send the distress message from one or more locations that have path loss values less than or equal to a threshold λ. The UAV is equipped with a GPS device to use it for navigation, and it flies with a maximum speed of *S* m/s. The flight time is slotted into *T* time slots, where the duration of each slot is δ seconds. The time slot duration includes the time for the UAV movement and data transmission. The UAV receives an acknowledgment from the access point when the message is received successfully. Otherwise, the UAV adjusts its location and resends the message again. Without loss of generality, we assume that the access point receives the message sent by the UAV successfully if the message is sent from a location where the path loss value is less than or equals to λ.

### Channel Model

Let $l_{i}=\left(x_{i},y_{i},z_{i}\right)$ be a 3D vector representing the location *i* on the path loss map. Path loss of the link between the UAV and the receiving access point when they are located at location *i* and *j*, respectively, is given by [22,23]

(1) $$\begin{array}{c} {\mathrm{PL}}_{ij}=\mathrm{Pr}\left(\mathrm{LoS},\theta_{ij}\right)\;{\mathrm{PL}}_{\mathrm{LoS}}\left(i,j\right)\;+\mathrm{Pr}\left(\mathrm{NLoS},\theta_{ij}\right)\;{\mathrm{PL}}_{\mathrm{NLoS}}\left(i,j\right) \end{array}$$

where $\mathrm{Pr}(\mathrm{LoS},\theta_{ij})$ is the probability of Line of Sight (LoS) communication between the UAV and the receiving access point when they are located at *i* and *j*, respectively, and the elevation angle between them is $\theta_{ij}$. Similarly, $\mathrm{Pr}(\mathrm{NLoS},\theta_{ij})$ is the probability of the Non-Line-of-Sight (NLoS) link between the UAV and the receiving access point. ${\mathrm{PL}}_{\mathrm{LoS}}\left(i,j\right)$ and ${\mathrm{PL}}_{\mathrm{NLoS}}\left(i,j\right)$ are the LoS and NLoS path loss of the link between the UAV and the receiving access point when they are located at *i* and *j*, respectively.

$\mathrm{Pr}\left(\mathrm{NLoS},\theta_{ij}\right)=1-\mathrm{Pr}\left(\mathrm{LoS},\theta_{ij}\right)$, and $\mathrm{Pr}(\mathrm{LoS},\theta_{ij})$, ${\mathrm{PL}}_{\mathrm{LoS}}\left(i,j\right)$ and ${\mathrm{PL}}_{\mathrm{NLoS}}\left(i,j\right)$ are given, respectively, by [23]

(2) $$\begin{array}{c} \mathrm{Pr}\left(\mathrm{LoS},\theta_{ij}\right)=\frac{1}{1+a\;\exp[-b\;\theta_{ij}+ab]} \end{array}$$

(3) $$\begin{array}{c} {\mathrm{PL}}_{\mathrm{LoS}}\left(i,j\right)=\frac{{\left(4\pi f\right)}^{2}}{c^{2}}\;(∥l_{i}-l_{j}{∥}^{2})\;\eta_{\mathrm{LoS}} \end{array}$$

(4) $$\begin{array}{c} {\mathrm{PL}}_{\mathrm{NLoS}}\left(i,j\right)=\frac{{\left(4\pi f\right)}^{2}}{c^{2}}\;(∥l_{i}-l_{j}{∥}^{2})\;\eta_{\mathrm{NLoS}} \end{array}$$

where *a* and *b* are parameters selected based on the environment, *c* is speed of light, *f* is the system frequency, and $\eta_{\mathrm{LoS}}$ and $\eta_{\mathrm{NLoS}}$ are the excessive path loss for LoS and NLoS links, respectively.

When the UAV is located at location *i*, the path loss at that location is the minimum path loss value out of all possible path loss values associated with *B* access points. Therefore, we have

(5) $${\bar{\mathrm{PL}}}_{i}=\min\left({\mathrm{PL}}_{i1},\ldots ,{\mathrm{PL}}_{iB}\right)$$

where ${\bar{\mathrm{PL}}}_{i}$ is the minimum path loss value at location *i*.

## 3. Problem Formulation

Let $L_{ij}\left(t\right)$ be a binary variable equals to one only when the UAV moves from location *i* to location *j* during slot *t*. During each time slot, the UAV moves only between two different locations or hovers at the same location. Therefore,

(6) $$\sum_{i\in N}\sum_{j\in N}L_{ij}\left(t\right)\leq 1,\;\;1\leq t\leq T.$$

The UAV movement during each time slot is restricted by the maximum distance that can be traveled, which is Sδ. Therefore, we have

(7) $$L_{ij}\left(t\right)\;d_{ij}\leq S\;\delta ,\;\forall i,j\in N,1\leq t\leq T.$$

where $d_{ij}$ is the distance between location *i* and location *j*.

Let α be the place of the incident for the object attached to the UAV and β be the final place that the UAV should go to after delivering the distress messages. The UAV uses the following three constraints to plan its flight trajectory starting from location α and ending at location β. First, the UAV should start flying right after the accident happened, i.e.,

(8) $$\sum_{j\in N}L_{\alpha j}\left(\hat{t}\right)=1$$

where $\hat{t}$ is the time slot associated with the accident. Moreover, the UAV goes to the final location, β, after delivering the distress messages and stays there, i.e.,

(9) $$\sum_{t=1}^{T}\sum_{i\in N}L_{i\beta }\left(t\right)=1$$

For a location *j* other than α and β, the UAV moving from location *i* to *j* during slot *t* must move from *j* to location *k* during the next time slot. Therefore, we have

(10) $$\sum_{i\in N}L_{ij}\left(t\right)-\sum_{k\in N}L_{jk}\left(t+1\right)=0,\forall j\in N\backslash \left(\alpha \cup \beta \right),1\leq t\leq T.$$

Let I(t) be an indicator function equals to one only when the UAV is located at a point in the path loss map associated with a path loss value less than or equals to λ, i.e.,

(11) $$\begin{array}{c} I\left(t\right)=\left\{\begin{array}{c} 1,\;\mathrm{if}\;P_{L}\left(t\right)\leq \lambda \\ 0,\;\mathrm{otherwise}. \end{array}\right. \end{array}$$

where $P_{L}\left(t\right)$ is the path loss value associated with the location of the UAV during slot *t*. Note that $\frac{P_{L}\left(t\right)}{\lambda }\leq 1$ when I(t)=1 and $1<\frac{P_{L}\left(t\right)}{\lambda }$ when I(t)=0. We define PL(t) by

(12) $$P_{L}\left(t\right)=\sum_{i\in N}\sum_{j\in N}L_{ij}\left(t\right)\;{\bar{\mathrm{PL}}}_{j}+\left(1-F(t)\right)\;M$$

where *M* is a large number and F(t) is described as follows:

(13) $$\begin{array}{c} F\left(t\right)=\left\{\begin{array}{c} 1,\;\mathrm{when}\;\mathrm{the}\;\mathrm{UAV}\;\mathrm{is}\;\mathrm{flying}\\ 0,\;\mathrm{otherwise}. \end{array}\right. \end{array}$$

From the description of F(t), we have

(14) $$F\left(t\right)=\sum_{i\in N}\sum_{j\in N}L_{ij}\left(t\right),\;\;1\leq t\leq T.$$

To replace the indicator function in (13) by constraints, we add the following two constraints:

(15) $$1-I\left(t\right)<\frac{P_{L}\left(t\right)}{\lambda },\;1\leq t\leq T.$$

and

(16) $$\frac{P_{L}\left(t\right)}{\lambda }\leq 1+\left[1-I(t)\right]\;M,\;1\leq t\leq T.$$

From constraint (15) and (16), I(t)=0 when $1<\frac{P_{L}\left(t\right)}{\lambda }\leq 1+M$, and I(t)=1 when $0<\frac{P_{L}\left(t\right)}{\lambda }\leq 1$.

The UAV tracks the acceleration of the object attached to it until it accedes the threshold π. Then, it detaches itself from the object and starts its mission in delivering the distress messages. Let a(t) be the maximum acceleration of the object attached to the UAV during time slot *t*. We define a binary variable, A(t), which equals one only when a(t) accedes the threshold π, as follows:

(17) $$\begin{array}{c} A\left(t\right)=\left\{\begin{array}{c} 1,\;\mathrm{if}\;\pi \leq a(t)\\ 0,\;\mathrm{otherwise}. \end{array}\right. \end{array}$$

where $1\leq \frac{a(t)}{\pi }$ when A(t)=1 and $\frac{a(t)}{\pi }<1$ when A(t)=0. To replace the indicator function in (17) by constraints, we add the following two constraints:

(18) $$A\left(t\right)\leq \frac{a(t)}{\pi },\;1\leq t\leq T.$$

and

(19) $$\frac{a(t)}{\pi }<1+A\left(t\right)\;M,\;1\leq t\leq T.$$

Then, we introduce the binary variable $\bar{A}\left(t\right)$ which equals to one only when the acceleration of the attached object acceded the threshold π during current or a previous time slot, i.e.,

(20) $$\begin{array}{c} \bar{A}\left(\bar{t}\right)=\left\{\begin{array}{c} 1,\;\mathrm{if}\;\exists \;A\left(t\right)=1\;s.t.\;t\leq \bar{t}.\\ 0,\;\mathrm{otherwise}. \end{array}\right. \end{array}$$

From (20), we introduce the following two constraints:

(21) $$A\left(t\right)\leq \bar{A}\left(\bar{t}\right),\;t\leq \bar{t}.$$

and

(22) $$\bar{A}\left(t\right)\leq \sum_{\bar{t}=1}^{t}A\left(\bar{t}\right),\;1\leq t\leq T.$$

Constraint (21) sets $\bar{A}\left(\bar{t}\right)$ to one when there is *t*, where $t\leq \bar{t}$, such that A(t)=1. On the other hand, constraint (22) sets $\bar{A}\left(t\right)$ to zero when there is no $\bar{t}$, where $\bar{t}\leq t$, such that $A(\bar{t})=1$. When the acceleration of the object attached to the UAV does not exceed the acceleration threshold π, then the UAV should not fly. Consequently, the variable $L_{i,j}\left(t\right)$ is set to zero. Thus,

(23) $$L_{i,j}\left(t\right)\leq \bar{A}\left(t\right),\;\forall i,j\in N,1\leq t\leq T.$$

Let γ be the minimum number of slots required by UAV to send the distress messages. Therefore, the UAV needs to include in its flying trajectory a minimum of one to γ nodes in the path loss map such that the path loss at these node is less than or equals to λ. The UAV transmits the distress messages over γ time slot and from one to γ locations. Let $T^{x}\left(t\right)$ be a binary variable defined as follows:

(24) $$\begin{array}{c} T^{x}\left(t\right)=\left\{\begin{array}{c} 1,\;\mathrm{if}\;\mathrm{the}\;\mathrm{UAV}\;\mathrm{sends}\;a\;\mathrm{distress}\;\mathrm{message}\\ \;\;\mathrm{during}\;\mathrm{time}\;\mathrm{slot}\;t.\\ 0,\;\mathrm{otherwise}. \end{array}\right. \end{array}$$

As the UAV should send γ messages to the emergency center, we have

(25) $$\sum_{t=1}^{T}T^{x}\left(t\right)=\gamma$$

Moreover, the UAV does not send a distress message during time slot *t* unless two conditions are satisfied: (1) the path loss value from the location of the UAV during slot *t* is less than or equals to the threshold λ and (2) the acceleration of the object attached to the UAV reached the threshold π during slot $\hat{t}$, such that $\hat{t}\leq t$. Accordingly, we add the following constraints:

(26) $$T^{x}\left(t\right)\leq I\left(t\right)\;\bar{A}\left(t\right),\;1\leq t\leq T.$$

Constraint (26) is nonlinear because the product of I(t) and $\bar{A}\left(t\right)$ is nonlinear. To linearize constraint (26), we introduce a new binary variable, $\Phi \left(t\right)=I\left(t\right)\;\bar{A}\left(t\right)$, and replace (26) by the following constraints:

(27) Φ(t)≤I(t),1≤t≤T.

(28) $$\Phi \left(t\right)\leq \bar{A}\left(t\right),\;1\leq t\leq T.$$

(29) $$I\left(t\right)+\bar{A}\left(t\right)-1\leq \Phi \left(t\right),\;1\leq t\leq T.$$

In our optimization problem, we have three objectives with different priorities ordered as follows: (1) minimizing the distress messages delivery time, (2) minimizing the UAV mission completion time and (3) minimizing the UAV traveled distance, which is related to the energy consumption of the UAV. As our objectives are prioritized, we can use the weighted sum method to formulate our objective function. Therefore, the formulation of our optimization problem is given by

(30) $$\begin{array}{c} \mathrm{Minimize}\;w_{1}\Omega^{D}+w_{2}\Omega^{F}+w_{3}\sum_{t=1}^{T}\sum_{i\in N}\sum_{j\in N}L_{ij}\left(t\right)d_{ij} \end{array}$$

**Subject to:**

Constraints (6)–(10), (12), (14)–(16), (18), (19), (21)–(23), (25) and (27)–(29).

(31) $$T^{x}\left(t\right)\;t\leq \Omega^{D},\;1\leq t\leq T.$$

(32) $$L_{i\beta }\left(t\right)\;t\leq \Omega^{F},\;\forall i\in N,1\leq t\leq T.$$

(33) $$0\leq \Omega^{D},\Omega^{F},\;\Omega^{D},\Omega^{F}\in Z.$$

(34) $$\begin{array}{cc} L_{ij}\left(t\right),I\left(t\right), & F\left(t\right),A\left(t\right),\bar{A}\left(t\right),\Phi \left(t\right),T^{x}\left(t\right)\in \left\{0,1\right\},\\ \; & \forall i,j\in N,1\leq t\leq T. \end{array}$$

$\Omega^{D}$ and $\Omega^{F}$ represent the number of used time slots to deliver the distress messages and complete the UAV mission, respectively. $w_{1}$, $w_{2}$ and $w_{3}$ are weights used to make a priority of a certain objective over another, where $w_{3}\ll w_{2}\ll w_{1}$. $\Omega_{D}$ and $\Omega_{F}$ are positive integer variables used in the objective function and the constraints (31) and (32) to minimize the maximum time slot indices associated with delivering the distress messages and completing the UAV mission.

## 4. Problem Solution

The optimization problem in Section 3 is in a form of Integer Linear Program (ILP), which is known to be NP-Complete [24]. To solve our problem in a polynomial time, we propose a Prioritized Multi-objective UAV Trajectory Optimization algorithm, as described in Algorithm 1. The UAV’s first and second priorities are delivering the distress messages and finishing the mission completion time, respectively, as soon as possible. The third priority is to go to its final location over the shortest path to safe the energy of the UAV’s battery. After successfully achieving these three goals, the UAV can reduce the time to dispatch the distress messages and the time of its active operation.
**Algorithm 1** Prioritized Multi-objective UAV Trajectory Optimization.  1:Set $N^{current}$ to the node ID associated with accident location.  2:Set $N^{next}$ to the node ID associated with the closest location that has an access to a communication service.  3:Find the shortest path, $P_{1}$, from $N^{current}$ to $N^{next}$.  4:Set $N^{current}=N^{next}$.  5:$P_{2}=\phi$.  6:**for** z=2⋯γ **do**  7:    **if** ∃$n\in Neighbor(N^{next})$ s.t. ${\bar{\mathrm{PL}}}_{n}\leq \lambda$ and *n* is the closest to the final location among all neighboring nodes and $N^{current}$ **then**  8:       Add *n* to $P_{2}$.  9:       $N^{next}=n$.10:  **else**11:      $P_{2}=N^{current}$.12:      Exit the loop.13:  **end if**14:**end for**15:$N^{current}=N^{next}$.16:Find the shortest path, $P_{3}$, from $N^{current}$ to the final location.17:Set the UAV trajectory path, *P*, to ($P_{1}\cup P_{2}\cup P3$).

The input to Algorithm 1 is a weighted graph, G, generated from the path loss map. The graph consists of all nodes in the path loss map, and there is an edge between any pair of adjacent nodes if the distance between them can be traveled by the UAV within one time slot. The weight of each edge is the UAV flying time between the pair of nodes associated with this edge. We utilize Dijkstra’s algorithm to calculate the shortest path between any pair of source and destination nodes.

In step 1 of Algorithm 1, we denote the location of the UAV after the accident happened by $N^{current}$. Then, the UAV searches for the closest node in the graph associated with a path loss value less than or equals to λ. We denote this node by $N^{next}$, as shown in step 2. The UAV calculates the shortest path, $P_{1}$, from $N^{current}$ to $N^{next}$ and flies to location $N^{next}$. Next, the UAV starts sending its distress message to the emergence center. The UAV set $N^{current}=N^{next}$ as shown in step 4.

Once the UAV reaches to a communication network access point, it can stay there until sending all distress messages then goes to its final location. However, the UAV can reduce its mission time by jointly sending the distress messages while going to its final location. In other words, the UAV can send its first distress message at $N^{next}$ node and send the remaining messages in the beginning of the return path to its final location. Accordingly, the UAV flies to a neighboring node to $N^{next}$ if the following conditions are met: (1) the neighboring node, *n*, satisfies the condition ${\bar{\mathrm{PL}}}_{n}\leq \lambda$ and (2) the location of the neighboring node, *n*, is closer to the final location. The UAV repeats the same process to send the subsequent distress messages until all messages are sent. If the two conditions are not met, the UAV stays at $N^{next}$ until sending all distress messages as described in the steps 5–14. Finally, the UAV flies to its final location and finishes its mission. The union of the three derived paths forms the UAV trajectory, as described in the steps 16–17.

### Time Complexity Analysis

In some steps of Algorithm 1, we find the shortest path between a pair of points using Dijkstra’s algorithm. The time complexity of finding the shortest path between two points using Dijkstra’s algorithm is $O\left(\left|E\right|+\left|V\right|\log_{2}\left|V\right|\right)$, where |E| and |V| are the number of edges and nodes in the graph, respectively. Let *K* be the maximum number of neighboring nodes connected to each node in the graph G. It is shown in step 7 of Algorithm 1 that the algorithm goes over *K* neighboring nodes to node $N^{next}$, in worst case, to search for the next candidate destination. This process is repeated for (γ−1) times in the worst case. Accordingly, the time complexity of Algorithm 1 is $O\left((|E|+|V|\log_{2}|V|)\;K\left(\gamma -1\right)\right)$.

## 5. Simulation Results

**Figure 4:**
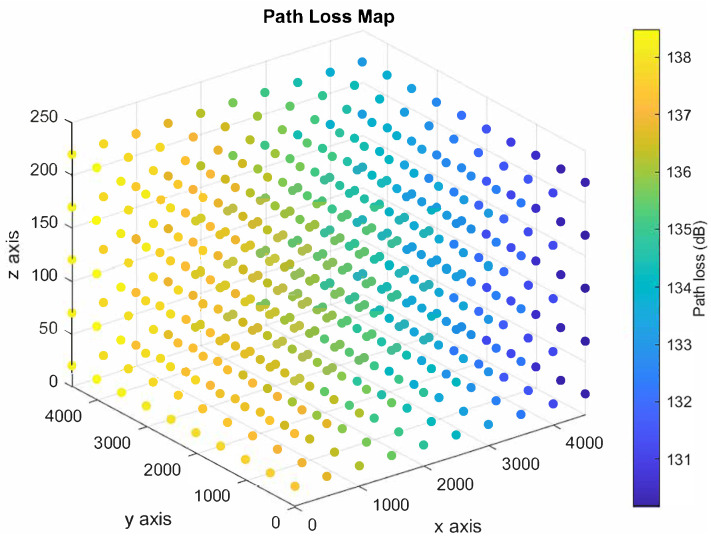
Dense path loss map.

**Figure 5:**
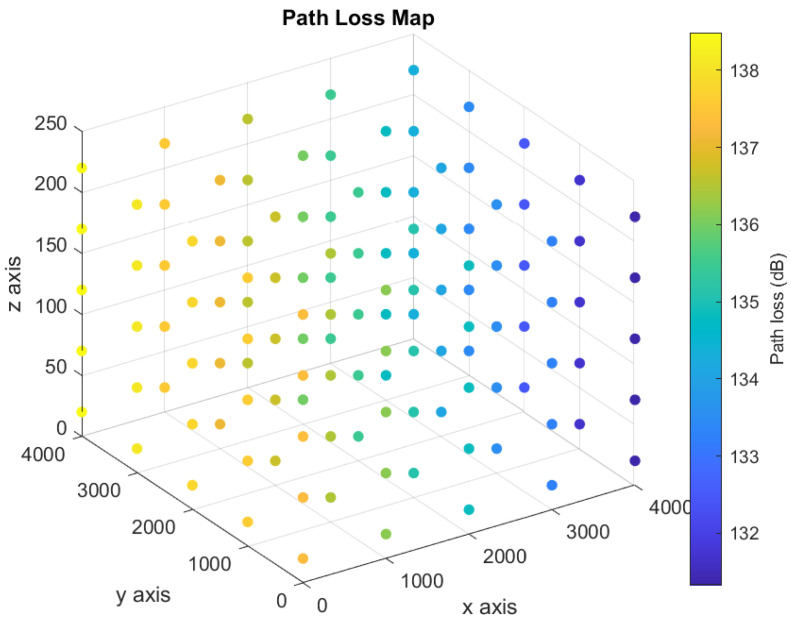
Sparse path loss map.

We consider a UAV serving as an automatic emergency dispatcher for a vehicle located at a coverage hole. Two base stations are located far away from the UAV, where their locations in the three-dimensional spaces are (8000, 0, 20) and (8000, 8000, 20). Based on the location of the base stations, we derive the path loss map. We use a three-dimensional space, 5000 m × 5000 m × 250 m, to model the path loss map. To study the effect of the number of points in the path loss map on the computational time, we consider two different grid sizes: (1) dense grid and (2) sparse grid. The dense grid, shown in Figure 4, consists of 500 path loss points (10 × 10 × 5 points, with a vertical/horizontal spacing of 50/1000 m). On the other hand, the sparse grid, shown in Figure 5, consists of 125 path loss points (5 × 5 × 5 points, with a vertical/horizontal spacing of 50/500 m).

The time is slotted into 20 time slots, with slot durations of 30 and 60 s when the path loss grid is dense and sparse, respectively. We assume that the vehicle served by the UAV faces an accident at location 1 and during the beginning of time slot 5. At time slot 5, the acceleration becomes 20 m/s^2^, which is above the threshold value 10 m/s^2^, i.e., π = 10 m/s^2^. The UAV should dispatch the distress messages and go to its final location. Unless specified otherwise, Table 1 shows the parameters used in our simulations.

### 5.1. Performance versus Path Loss Map Density

First, we investigate the effect of the path loss map density (number of points) on the accuracy of the optimal solution and the computational time required to get the optimal solution. As shown in Table 2, we can get a better solution when we adopt the dense grid. The dense gird has a larger number of nodes distributed over the 3D space, which allows the UAV to have more shorter path options. Therefore, the dense grid provides more flexibility for the UAV to reach to a location with low path loss value and to reach to the final location in a shorter distance and time. On the other hand, it is shown in Table 2 that adopting the sparse grid can reduce the computational time significantly although it leads to degrading the obtained solution. Therefore, there is a trade-off between getting a more accurate solution and reducing the computational time. Hence, we should select the path loss map density based on the UAV computational power and our flexibility of getting a less accurate result.

### 5.2. Optimal versus Algorithm 1’s UAV Routing Trajectories

**Figure 6:**
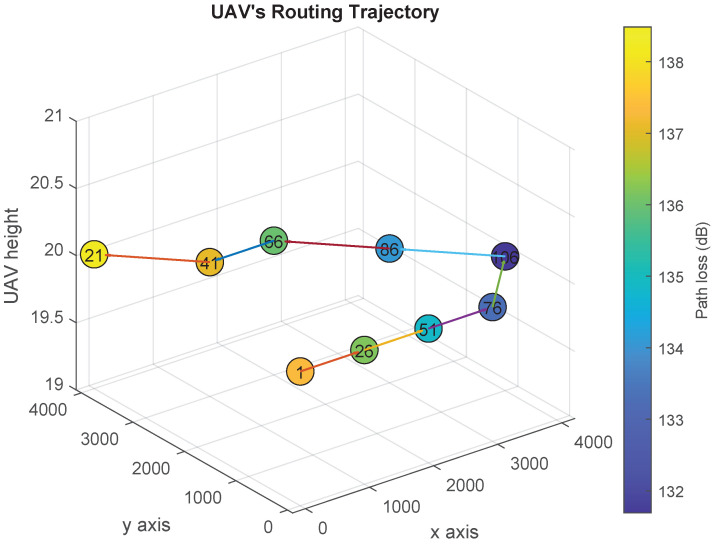
Optimal UAV’s trajectory design over the path loss map (Scenario 1).

**Figure 7:**
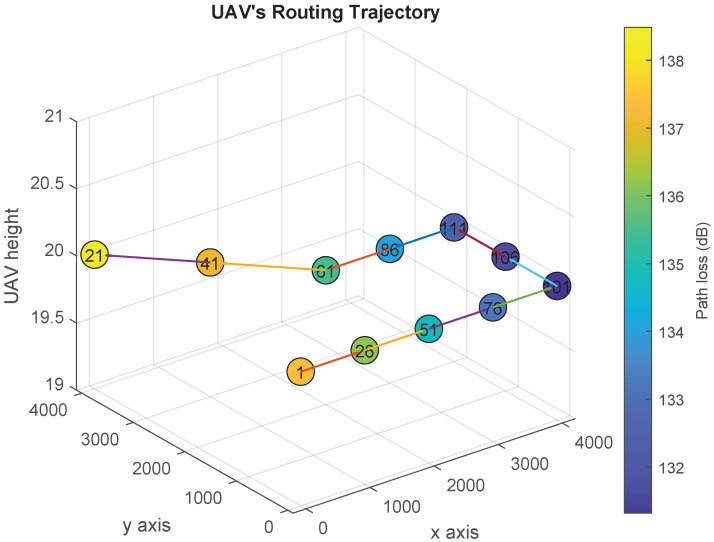
UAV’s trajectory design over the path loss map using Algorithm 1 (Scenario 1).

**Figure 8:**
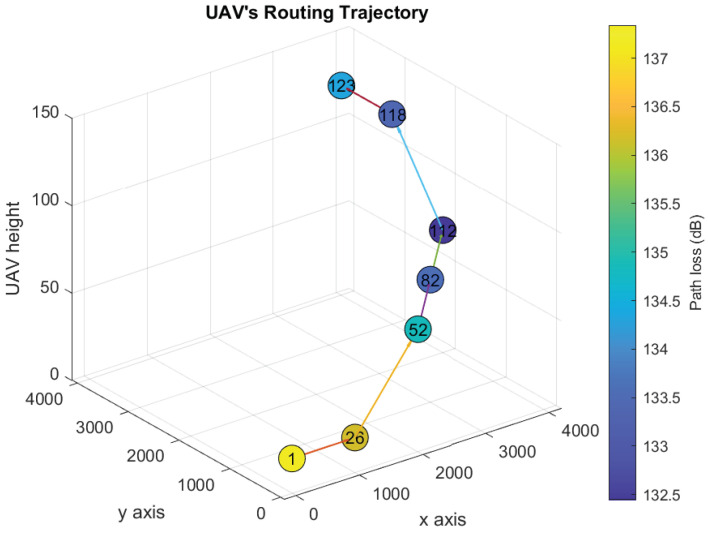
Optimal UAV’s trajectory design over the path loss map (Scenario 2).

**Figure 9:**
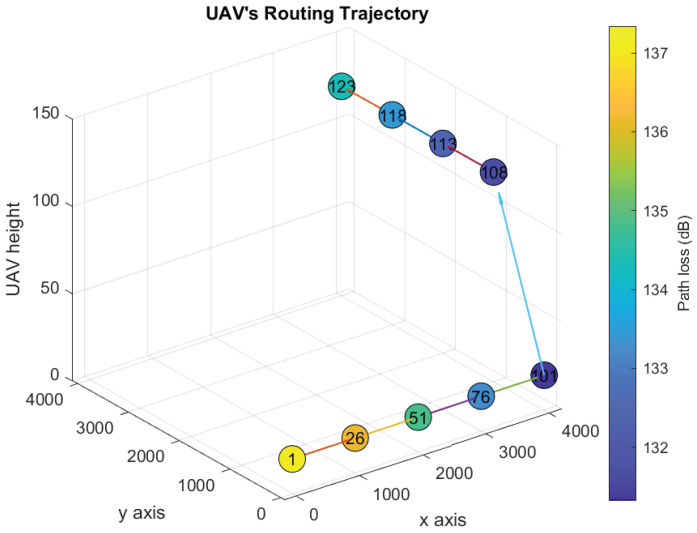
UAV’s trajectory design over the path loss map using Algorithm 1 (Scenario 2).

Figure 6, Figure 7, Figure 8 and Figure 9 show the UAV’s trajectories for scenarios 1 and 2, when we solve the original optimization problem and Algorithm 1. We assume that the threshold λ=133 dB. Therefore, the UAV flies to one or more locations associated with path loss vales less than or equal to 133 dB and sends the distress message from them. In scenarios 1 and 2, the UAV goes to locations 21 and 123, respectively, after delivering the distress messages.

In Figure 6, we present the optimal UAV trajectory of scenario 1. After the acceleration of the UAV exceeds the threshold π, it searches for the closest location that has an access to the communication network (i.e., closest location *j* such that ${\bar{\mathrm{PL}}}_{j}\leq \lambda$). Accordingly, the UAV goes from location 1 to location 106, and stays there for γ slots to sends the distress message. Then, it flies to the final location to node 21. Similarly, Figure 7 shows the UAV trajectory generated using Algorithm 1. The UAV flies to location 101 and sends the first distress message, then flies to location 106 and 111 to sends the rest of the distress messages. Finally, it goes back to its final location. In both solutions, the UAV finishes delivering the distress message and goes back to its final location by the end of slot 11 and 14, respectively.

The UAV’s trajectories of scenario 2 derived after solving the original optimization problem and Algorithm 1 are shown in Figure 8 and Figure 9, respectively. Scenario 2 is similar to scenario 1 except that the final location is at location 123. In both solutions, the UAV delivers the distress messages and goes back to the final location by the end of time slot 11 and 12, respectively. Although Algorithm 1 solution is similar to the optimal, the computational time is reduced significantly, as shown in Table 3.

### 5.3. The Effect of the Path Loss Threshold Value on the Performance

**Figure 10:**
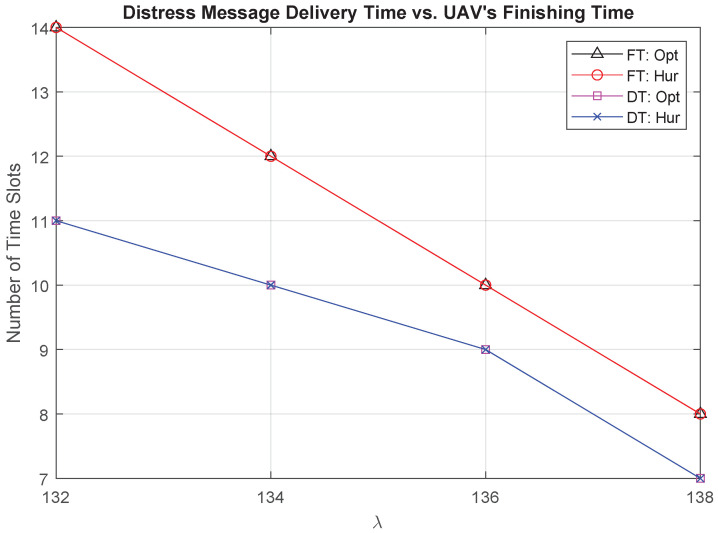
Finishing time vs. delivery time vs. λ.

We present the relationship between the threshold λ and the distress messages Delivery Time (DT) and the UAV Finishing Time (FT) in Figure 10. DT is the time slot during which the UAV delivers all distress messages, where FT is the time slot that the UAV finishes its task and returns to its final location. It is shown that the solution of Algorithm 1 is similar to the optimal under different values for λ. As we increase the value of λ, the UAV has more chances to reach to a closer location with an access to a communication network. Therefore, the UAV can deliver the distress messages and return to its final location within a shorter time. Moreover, the gap between DT and FT decreases when λ increases as the UAV will have more options to go to locations in the direction of the final location to deliver the distress messages.

**Figure 11:**
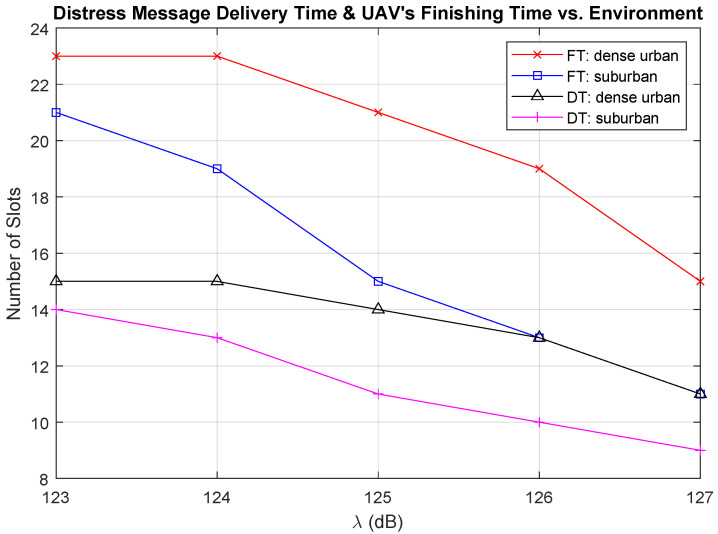
Finishing time vs. delivery time vs. the environment.

We present the effect of the environment on DT and FT in Figure 11. Given a certain location in the path loss map, the path loss value in the dense urban environment is higher compared to the path loss value in the suburban environment. Therefore, DT and FT are shorter in the suburban environment. The gap between the solutions under these different environments is large, where the FT in the suburban environment is similar to DT in the dense urban environment. From Figure 10 and Figure 11, we observe that the value of the threshold λ and the environment where the UAV flies can significantly affect the required time to send the distress messages. The selection for the value of λ depends on the quality of service requirement, the environment where the UAV flies, and the delay tolerance for delivering the distress messages.

## 6. Conclusions

In this paper, we proposed a UAV-based method for conveying the distress messages from coverage holes to the emergency center. First, we model the problem using a three-dimensional path loss map. Then, we formulate a mathematical model to minimize the time required to deliver the distress message and complete the UAV’s mission time. We proposed a heuristic algorithm to solve our problem more efficiently. Additionally, we investigate our scheme and simulate the proposed algorithm under different scenarios. The effect of the path loss map density is studied, where the dense map allows finding more accurate results. On the other hand, sparse path loss map simplifies the routing problem and allows solving the problem in a shorter time. Moreover, we verify the advantage of our proposed heuristic algorithm where it finds the solution with a significant reduction in the computational time. Our results show that the value of the threshold λ has a great impact on the distress messages delivery time. Therefore, a higher quality of service requirements for delivering the distress messages results in more delay in the delivery.

## 7. Patents

The US patent titled “Emergency unmanned aerial vehicle and method for deploying an unmanned aerial vehicle” with number 9834306 results from this paper.

## Figures and Tables

**Table 1:** Simulation parameters.

Parameter	Value	Parameter	Value
*N*	125, 500	*f*	2 GHz
δ	30, 60 s	*c*	$3\times 10^{8}$ m/s
*T*	20	*a*	11.25, 8.96
*S*	23 m/s	*b*	0.06, 0.04
*B*	2	γ	3
$\eta_{\mathrm{LoS}}$	0.1, 1.6 dB	λ	132–138 dB
$\eta_{\mathrm{NLoS}}$	21, 23 dB	π	10 m/s^2^

**Table 2:** Computational vs. delivery time.

	Dense Grid	Sparse Grid
Slot duration (s)	30	60
Computational time (s)	1505	13
Slots to deliver the message	11	9
Slots to return to final location	16	12
Time to deliver the message (s)	330	540
Time to return to final location	480	720

**Table 3:** Computational time.

	Scenario 1	Scenario 2
Time to get the optimal solution	57 s	60 s
Time to get Algorithm 1 solution	0.023 s	0.023 s
